# Advances in Cleft Lip and Palate Surgery

**DOI:** 10.3390/medicina59111932

**Published:** 2023-11-01

**Authors:** Mario A. Aycart, Edward J. Caterson

**Affiliations:** Department of Surgery, Division of Plastic and Reconstructive Surgery, Nemours Children’s Health-Delaware, 1600 Rockland Road, Wilmington, DE 19803, USA; edward.caterson@nemours.org

**Keywords:** cleft lip, cleft palate, presurgical infant orthopedics

## Abstract

Cleft lip with or without cleft palate is one of the most common congenital malformations, with an average prevalence of 1 in 1000 live births. Cleft lip and/or palate is incredibly phenotypically diverse, with constant advancements and refinements in how we care for patients. This article presents an in-depth review of the latest advances and current evidence in cleft lip and palate surgery. This includes presurgical infant orthopedics, perioperative practice patterns including use of enhanced recovery after surgery (ERAS) protocols, patient-reported outcome measures, and the latest adjuncts in cheiloplasty and palatoplasty.

## 1. Introduction

The history of cleft lip and palate surgery is rich with innovation and there is a constant push to provide optimal form and function for our patients. This has led to centuries of refinements that have challenged cleft surgeons to improve upon the work performed by those before them. The often-quoted Dr. Ralph Millard captured the essence of the field that still resonates today, “Semper investigans, nunquam perficiens. Always searching, never quite achieving perfection” [1].

The purpose of this article is to provide an in-depth evidence-based review of the latest advances in cleft lip and palate surgery, including presurgical infant orthopedics, patient-reported outcome measures, perioperative practice patterns including use of enhanced recovery after surgery (ERAS) protocols, and the latest adjuncts in cheiloplasty and palatoplasty.

## 2. Pre-Surgical Infant Orthopedics

In 2019, the American Cleft Palate-Craniofacial Association (ACPA) completed a survey of multidisciplinary cleft teams; half of the teams offered presurgical infant orthopedics (PSIO), with nasoalveolar molding (NAM) comprising 88.2% and the Latham appliance comprising 14.7% [2]. Lip taping, however, is by far the simplest, least labor-intensive, and most cost-effective method of presurgical infant orthopedics and is often used when patients are not candidates for other forms of PSIO. Recently, there have been increasing reports regarding the use and efficacy in cleft care [3,4,5]. Initial reports date back to 1905, by G. V. I. Brown [6], with a more contemporary study by Dr. Robert Pool in 1994 reporting on their experience of 22 patients (17 unilateral and 5 bilateral) over 16 years achieving a 53% reduction in the size of the gap of the alveolar segments (12.4 mm to 5.8 mm) prior to definitive cheiloplasty [7]. More recently, there have been two randomized clinical trials (RCTs) noting the efficacy of presurgical lip taping in unilateral cleft lip and palate on positively changing the maxillary arch dimensions [3] and nasolabial aesthetics [5]. Comparing two of the most commonly used PSIO in NAM, lip taping has achieved similar results with regard to reductions in alveolar gap and nasolabial symmetry in two prospective studies [8,9]. In a separate retrospective study, at Washington University in St. Louis, NAM was compared to a passive alveolar molding plate plus lip taping with similar outcomes [4]. Noteworthy was that the observed burden of care was higher in families using NAM, as analyzed through number of patient appointments attended, treatment costs, and caregiver satisfaction surveys, when compared to passive alveolar molding and lip taping. No such comparative study exists examining the burden of care for NAM versus lip taping alone. While NAM has been criticized for its inconsistent efficacy regarding changes to nasal symmetry [10], high costs, and burden of care, more recent retrospective, single-institution mid- [11,12] and long-term outcome studies [13] have shown sustained, improved nasolabial aesthetics. A recent systematic review of 88 total studies supports the overall efficacy of NAM in short- to mid-term outcomes [14].

One particular advance in the field of PSIO and NAM is the potential for custom three-dimensional (3D) printing and the advent of digital orthodontics. This was introduced by Yu et al. as a method of computer-aided design/nasoalveolar molding or CAD/NAM using a 3D laser scanner and reverse engineering software for simplification of the model construction [15]. More recently, an RCT introduced a technique where all appliances were 3D-printed and virtually constructed, and without the need for manual appliance construction in patients with unilateral complete cleft lip and palate compared to no treatment [16]. The authors reported on the cost and material considerations with faster (20–30 min per patient) and more precise appliance construction where prototyping and fabrication advancements may lessen the burden of care and appointment times for families.

There are several options available for PSIO, and providers should carefully consider each patient and their social determinants of health and psychosocial support prior to recommending specific forms of PSIO to minimize morbidity and optimize patient outcomes.

## 3. Advances in the Perioperative Care of Cleft Lip and Palate Surgery

Perioperative practices for cleft care have harnessed the principles of enhanced recovery after surgery and quality improvement initiatives, with the goal of providing patient-centered, value-based care. An important aspect of these protocols is pain management, specifically opioid minimization and multimodal pain management. Other primary outcomes including decreasing hospital length of stay and time to initiating oral intake [17] are pertinent. In the case of cheiloplasty and palatoplasty, these metrics are often readily achieved with the adjunct use of intra-operative nerve blocks.

There have been several RCTs evaluating the efficacy of intra-operative infraorbital nerve blocks versus placebo in cheiloplasty, demonstrating significantly improved pain scores and time to analgesic failure [18,19,20,21]. In the case of palatoplasty, bilateral suprazygomatic maxillary nerve blocks have been shown to be efficacious and safe in a double-blinded RCT versus placebo [22]. This nerve block has also been compared versus traditional peripheral nerve blocks such as bilateral infraorbital nerve blocks in cheiloplasty and greater and lesser palatine nerve blocks and the nasopalatine nerve block in palatoplasty, and was shown to reduce intra-operative nalbuphine consumption and requirements for fentanyl, without differences in post-operative pain scores or complications [23]. A recent systematic review and meta-analysis of RCTs made the following recommendations for pain management following cleft lip and palate surgery [17]:

1. Preincisional nerve block with long-acting anesthetics for both cheiloplasty and palatoplasty.
2. For cheiloplasty, infraorbital nerve blocks should be performed with the addition of nasal blocks for cleft rhinoplasty.
3. For palatoplasty, nerve blocks include maxillary, sphenopalatine, or greater/lesser/nasopalatine. If a maxillary nerve block is performed, then a suprazygomatic approach is preferred.
4. Non-opioid alternatives, such as acetaminophen and non-steroidal anti-inflammatory medications, as part of multimodal pain control should be used.

The application of standardized clinical pathways in cleft care has also been shown to decrease length of stay and opioid consumption without concomitant readmissions or return to the emergency department [24]. Multidisciplinary quality improvement studies to decrease opioid use demonstrate the institutional efforts required to reprioritize goals and standardize goal setting between all key stakeholders. A recent study by Lee et al. highlighted the importance of a series of integrated, coordinated, multidisciplinary efforts including preoperative counseling, intra-operative pain management, and postoperative education and standardization of care to successfully reduce opioid consumption while maintaining length of stay and pain satisfaction scores in cleft care [25].

Increasing emphasis on providing efficient, value-based health care has prompted cleft teams to consider outpatient cheiloplasty for properly selected patients. There have been several studies reporting on experiences with overall positive outcomes in reducing length of stay without adversely affecting outcomes or readmissions [26,27,28,29]. The largest study included a retrospective review of six centers over seven years totaling 546 patients with an ambulatory surgery rate of 81 percent with no significant differences in emergency department visits or readmissions in a carefully selected cohort of patients. They noted that patients with comorbidities and syndromes were at increased risk for readmission and recommended admission for observation [28]. In a recent single-institution retrospective study, 226 patients with unilateral cleft lip and 58 patients with bilateral cleft lip were included, where 80% of the unilateral group and 56% of the bilateral group received ambulatory surgery with an average length of stay of 8 h, compared to 24 h preintervention. Patients requiring cardiac or airway monitoring, with history of prematurity and comorbidities, were chosen for overnight stay instead [30]. This study also showed the safety and feasibility of ambulatory cleft lip surgery, again, in the carefully selected patients.

## 4. Advances in Cleft Lip Surgery

Although much effort and progress has been made to benchmark all aspects of cleft care, there remains large variation in practice patterns [31]. Central to the care of the patient with a cleft lip and/or palate is cheiloplasty and whether and to what extent to perform primary rhinoplasty. Though the history of cleft lip repair dates back centuries and remains an enriching and fascinating topic [32], the focus of this article will be on advances and adjuncts regarding the two most commonly utilized techniques: rotation-advancement and straight-line repairs.

With the introduction of the anatomical subunit approximation technique by David Fisher in 2005 [33], there has been growing adoption of this technique for primary cheiloplasty and several comparative studies to the commonly performed rotation-advancement repair and its modifications. The latest ACPA survey in 2015 of 86 cleft surgeons reported that 38% had changed technique for unilateral cleft lip to the Fisher anatomical subunit repair, though 54% of respondents still utilized a Millard approach with primary rhinoplasty being performed 57% of the time for complete unilateral cleft lip [34]. Two studies have reported on single-surgeon experiences converting from rotation-advancement repair to Fisher repair and retrospectively evaluated their outcomes for each repair. There are two studies, in Patel et al. [35] and in Rohit Khosla’s case series [36], that detail a surgeon’s experience in their transition from extended Mohler repair to Fisher repair. The latter case series included 68 patients over seven years with 34 patients having undergone rotation-advancement repair and 35 the Fisher repair. Revision rates for scar contracture, hypertrophy, and widening decreased from 36% to 2.9%, though with a rise in rates of minor debulking of vermillion fullness from 6.1% to 37%. However, both studies can be critiqued for the important acquisition of surgical experience as a source of bias when evaluating such outcomes, as both began their careers with rotation-advancement repairs and then transitioned to a Fisher repair.

To address these shortcomings, Deshmukh et al. conducted a prospective, randomized, observer-blinded study on the comparative evaluation of aesthetic outcomes in unilateral cleft lip repair [37]. The study included 50 patients randomized to either Mohler or Fisher repairs, repaired by a single surgeon (blinded regarding which technique they were going to perform until the patient was taken to the operating room and intubated under general anesthesia), and postoperative photographs were evaluated by three laymen using the Surgical Outcomes Evaluation Scale with means adjusted by cleft severity into four grades (mild incomplete, incomplete, complete, and severe complete) using the Unilateral Cleft Lip Severity Index. In this study, the Fisher technique had a significantly (*p* = 0.0153) better mean aesthetic outcome than the Mohler technique. The mean cleft severity was 2.96, and the adjusted mean aesthetic outcome scores for the Fisher and Mohler techniques were 5.0512 and 4.3088, respectively. There have also been studies comparing aesthetic outcomes of the Fisher, Millard, or Mohler techniques using eye-tracking technology [38] and online crowdsourcing [39], with both studies showing superior results for the Fisher repair. While both techniques can attain excellent results, these studies may shed light on the recent trend of cleft surgeons transitioning from rotation-advancement repair to Fisher repair.

One interesting adjunct in cheiloplasty is that of the use of botulinum toxin. Injection of botulinum toxin into the orbicularis oris can reduce continuous muscle contraction and tension to create a more optimal environment for wound healing and therefore a more favorable scar. This has been investigated in two RCTs in patients with unilateral cleft lip, with the first by Chang et al., from Chang Gung Memorial Hospital, in a double-blind, randomized, vehicle-controlled, prospective trial of 60 patients, of which 59 completed the trial [40]. Patients were assessed using the Vancouver Scar Scale (VSS) and the Visual Analog Scale (VAS) using photographic measurements and subjective interpretations from expert and general public evaluators at 6-month follow-up. The results from this study showed that scars were objectively narrower and subjectively better (Visual Analog Scale only) when compared with the control group. Importantly, there were no complications such as infection, bleeding, dehiscence, oral incontinence, or feeding dysfunction. A second more recent study by Sonane et al. also devised a similar RCT enrolling 28 infants (with 22 patients completing the study) undergoing unilateral cleft lip repair rated by blinded experts at 6-month follow-up [41]. They also used the VSS, the VAS, and photographic scar width measurements. They also found improved VAS scores and narrower scar width with no differences in VSS compared to control group. The use of botulinum toxin appears to be a safe and promising efficacious adjunct in cheiloplasty, though this must be applied to the appropriate patient population, i.e., those most at-risk for hypertrophic scarring [42], which was followed in both RCTs.

## 5. Advances in Cleft Palate Surgery

Cleft palate surgery dates back centuries and there still exists significant variation in the technique and timing of palatoplasty and a lack high-quality data to evaluate long-term outcomes and evidence-based recommendations [43]. Herein, we highlight the latest advances in adjunctive techniques and modifications in palatoplasty to mitigate complications and optimize outcomes and highlight the most recent RCT from the Timing of Primary Surgery for Cleft Palate (TOPS) study group [44].

### 5.1. Timing of Palatoplasty

The timing of palatoplasty has long been a controversial topic. The ACPA recommends palatoplasty prior to 18 months of age [45], with most centers performing palatoplasty at between 6 and 14 months. However, there are data to suggest that late palatoplasty (<13–15 months of age) is associated with poorer long-term speech outcomes [46,47], though with less impact on midface growth disruption and maxillary arch constriction. The TOPS study group recently published an international, two-group RCT that assessed whether early palatoplasty at 6 months of age (266 patients) produced better speech outcomes than late palatoplasty at 12 months (255 patients) in medically fit, non-syndromic patients with an isolated cleft palate [44]. The primary outcome was velopharyngeal insufficiency (VPI) at 5 years of age with secondary outcomes including hearing, dental occlusion, and feeding. Surgery was performed with the use of the Sommerlad technique with all surgeons receiving in-person instruction from Mr. Brian Sommerlad with written descriptions and video presentations. The study concluded that early surgery was associated with better speech outcomes with 8.9% (21/235) VPI in the early-surgery group compared to 15% (34/226) in the late-surgery group. However, there was a very important post hoc analysis performed that showed that the incidence of secondary surgery was actually higher in the early-surgery group with 30 procedures in 27 children (9.7%) compared to 17 procedures in 16 children (5.9%). Once these patients were recategorized using a composite strategy estimand with respect to secondary surgery, the primary outcome of VPI between the two groups was much smaller and did not reach statistical significance—a risk ratio of 0.88 (95% confidence interval 0.60 to 1.28). Specifically, there were 41 children in the early-surgery group compared to 45 children in the late-surgery group. There were no differences at 3 and 5 years of age with regard to hearing sensitivity and middle-ear function, and growth and dentofacial development showed greater arch constriction in the early-surgery group with similar anthropometric outcomes at 5 years of age. While this study concludes that early surgery produced better speech outcomes at 5 years, the longer-term effects on dentofacial development, need for orthognathic surgery, and burden of care remain to be seen. Nonetheless, this study is a major accomplishment and a landmark study in cleft care.

### 5.2. Buccal Fat Pad Flaps

Buchman and colleagues described the use of buccal fat pad (BFP) flaps in palatoplasty and oronasal fistula repair for coverage of denuded hard palate (lateral relaxing incisions) and interposed between oral and nasal layers in areas of high tension [48]. The initial report was on 10 patients with primary palatoplasty and in 4 patients with oronasal fistulas with 12-week follow-up without postoperative oronasal fistulas. Since then, several other reports have followed that demonstrate overall positive outcomes. A recent literature review included 35 articles and 666 patients in primary and secondary palatoplasty using BFP flaps with 24 cases of oronasal fistula, 2 dehiscences, and 4 transient mucosal defects [49]. These 30 cases of complications comprised 4.5% of total cases. Donor site morbidity was noted to be minimal with epithelialization noted anywhere from 2 to 6 weeks with minor swelling resolving within the first week after surgery.

One particular concern is that of creating facial asymmetry from BFP harvest. Utilizing volumetric analysis with 3D photogrammetry, Bennett et al. did not demonstrate clinically or statistically significant facial asymmetry with unilateral BFP harvest using the patient as an internal control, with an average follow-up of 55 months [50]. Interestingly, an MRI study in a single patient showed viable bilateral BFP flaps with maintained position as evaluated using magnetic resonance imaging (supine position, and no sedation) at 5 years after primary palatoplasty and bilateral BFP flaps [51].

Lastly, one important use for which BFP was originally advocated was the coverage of a denuded or lateral raw surface of the hard palate to help mitigate transverse maxillary growth restriction. CC Lo et al., from Chang Gung Memorial Hospital, provided an excellent long-term 3D-imaging-assisted study to assess the impact of BFP flaps on posterior transverse maxillary growth [52]. Their study consisted of 54 patients with unilateral cleft lip and palate undergoing modified Furlow palatoplasty at 9–10 months of age who received BFP flaps (n = 22) or Surgicel (n = 32), and a third group including unilateral cleft lip and alveolus only (n = 24) as a “non-palatoplasty” group was included as a comparison. A cone-beam computed tomography (CBCT) scan was obtained as work-up prior to alveolar bone graft at 9 years of age. The BFP flaps had statistically significant wider total transverse maxillary dimensions compared to Surgicel as evaluated through standardized measurements in CBCT, while palatal length was equivalent between the BFP flaps and Surgicel groups and statistically significantly less than the non-palatoplasty group. Given their ease of harvest, minimal donor site morbidity (short term), vascularized autologous nature, and potential to ameliorate transverse maxillary development, the BFP flaps have become an increasingly utilized adjunct in primary and secondary palatoplasty.

### 5.3. Buccal Myomucosal Flaps

Within a similar light, the literature on augmentative palatoplasty with the use of buccal myomucosal flaps (BMMFs) in primary and secondary palatoplasty has continued to gain traction since its early description as an addition to Furlow palatoplasty over two decades ago by Fisher and Mann [53]. More recently, Mann and colleagues have shown excellent speech and fistula outcomes in their series of 505 consecutive cases using Furlow palatoplasty with and without BMMF. Of these patients, there were 319 that were treated with Furlow palatoplasty plus BMMF with an average length of follow-up of 7.76 years. The addition of BMMF were noted to be particularly useful for wider (>10 mm) and more complex clefts with similar speech outcomes and postoperative oronasal fistulae [54].

Though described as early as 1969 [55], Ian Jackson and colleagues published one of the earlier reports on the use of BMMF in secondary palatoplasty for velopharyngeal dysfunction (VPD) and oronasal fistula management in 22 patients, with 77% of patients displaying improved speech quality [56]. Recently, there have been increasing reports of BMMF for velopharyngeal dysfunction. This has been described as an interposition of flaps into a transverse defect for palatal lengthening after previous unsuccessful treatments for VPD [57] and as part of nuanced algorithms combining BMMF with Furlow palatoplasty or BMMF alone for palatal lengthening [58], with excellent results without obstructive sleep apnea with average follow-ups of 15 months and 8.9 months, respectively. The most recent study by Chiang et al. reported on 25 patients undergoing BMMF for VPD at a median of 7.1 years after primary palatoplasty. Sixteen patients (64%) had undergone additional procedures such as repair of oronasal fistulae and palatal lengthening, and three had undergone posterior pharyngeal flaps that were taken down prior to BMMF. In this study, BMMF was combined with Furlow palatoplasty in 60% of cases versus BMMF alone, with an average time to buccal flap division of 3.7 months with 88% of patients achieving functional speech and no reports of obstructive sleep apnea with an average follow-up time of 1.7 years [59]. Subgroup analysis between these two groups did not have statistically significant differences with regard to postoperative velar closure or speech scores, though with such small groups (15 combined Furlow palatoplasty versus 10 BMMF alone) their sample size may not have been powered to detect a difference. BMMF flaps hold great promise as an alternative to pharyngoplasty and maintain a more anatomic and dynamic velum to produce normal speech.

## 6. Outcome Measurements

**Figure 1:**
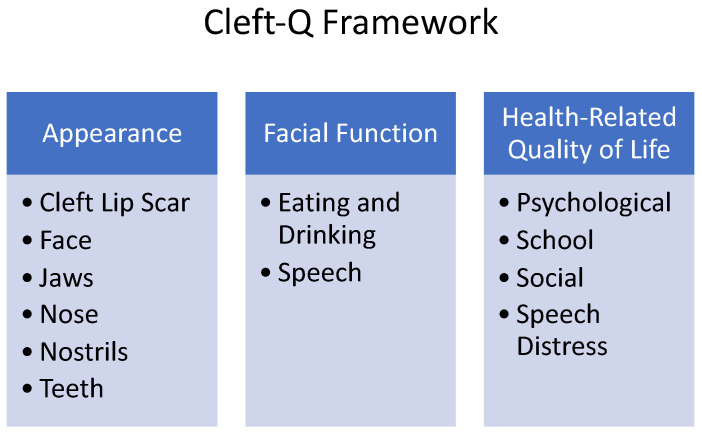
The Cleft-Q framework with its 12 scales and three domains. Cleft-Q provides the flexibility to choose the subset of scales best suited for particular situations in both research and clinical use.

**Figure 2:**
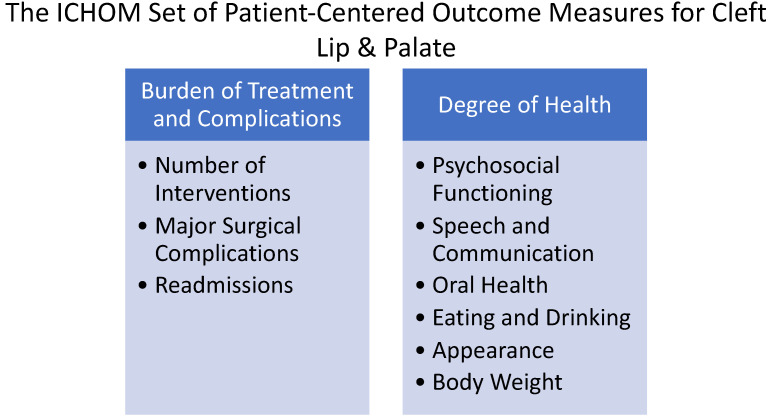
The ICHOM set of measures should be collected from patients, clinicians, and administrative sources and have suggested timings and age categories for measurement, including a baseline in addition to the following time points: 3 months, 5 years, 8 years, 12 years, 22 years, and within 30 days following the date of the operation.

Patient-reported outcome measures (PROMs) are becoming increasingly popular in all aspects of medicine and surgery, including plastic surgery [60]. PROMs are tools that can be used to assess the impact of surgery (or other procedures) on a patient’s or caregiver’s quality of life. They turn the subjective into measurable and actionable data and allow for a quantifiable objective evaluation. Within the field of cleft lip and palate, there are two main tools utilized today. CLEFT-Q is a condition-specific PROM, developed and validated for children and adolescents aged 8 to 29 years with cleft lip and/or palate [61]. CLEFT-Q measures three main domains, with each domain composed of one or more independently functioning scales (see Figure 1). The International Consortium for Health Outcomes Measurement (ICHOM) has also produced a standard set of standardized outcome measures [62] that is also commonly used by cleft teams for the comprehensive appraisal of cleft care (see Figure 2).

CLEFT-Q continues to undergo translation and validation to other languages worldwide [63,64,65,66]. Multiple studies have demonstrated the validity and utility of CLEFT-Q [67] and the ICHOM standard set to the extent that, at this point, research is moving beyond content validity and focusing on the feasibility of implementation and verifying scales for their utility in clinically significant decision making. As more centers incorporate and attempt to implement these tools in their practice, the feasibility of implementation and data collection may become a challenge. Recent work sheds light on this problem and common barriers to and facilitators of implementation have been identified through the work of four cleft teams in Europe and North America as pilot centers in implementing the ICHOM standard set [68]. With regard to the adoption of the ICHOM standard set, the three main themes identified were creating importance and urgency, aligning motivation and priorities through regular meetings, and securing resources. Institutional support and financial resources are vital to effective adoption and implementation, which can be a lengthy and labor-intensive process. Multisite collaboratives are also available to help facilitate implementation, such as ACCQUIREnet led by Duke University, which is making its REDcap electronic system for data collection available to sites who join the collaborative.

## 7. Conclusions

In conclusion, the field continues to evolve and improve. There is constant pressure that we as cleft surgeons place on ourselves and our colleagues to seek the unattainable perfection. Over the next several years, we will see continued evolution of cleft care as adjacent specialty surgical services will continue to participate in the primary surgical treatment of cleft lip and palate. Therefore, this relentless pursuit of excellence is paramount to continued plastic and craniofacial surgery innovation in this area of expertise. We as authors and fellow surgeons can think of no more impactful and gratifying pursuit in the field of surgery.

## Data Availability

Data sharing not applicable. No new data were created or analyzed in this study. Data sharing is not applicable to this article.
